# The Value of Laboratory Parameters for Anemia, Renal Function, Systemic Inflammation and Nutritional Status as Predictors for Outcome in Elderly Patients with Head-and-Neck Cancers

**DOI:** 10.3390/cancers12061698

**Published:** 2020-06-26

**Authors:** Alexander Rühle, Erik Haehl, Hélène David, Tobias Kalckreuth, Tanja Sprave, Raluca Stoian, Constantinos Zamboglou, Eleni Gkika, Andreas Knopf, Anca-Ligia Grosu, Nils Henrik Nicolay

**Affiliations:** 1 Department of Radiation Oncology, University of Freiburg—Medical Center, Robert-Koch-Str. 3, 79106 Freiburg, Germany; alexander.ruehle@uniklinik-freiburg.de (A.R.); erik.haehl@uniklinik-freiburg.de (E.H.); helene.david@uniklinik-freiburg.de (H.D.); tobias.kalckreuth@uniklinik-freiburg.de (T.K.); tanja.sprave@uniklinik-freiburg.de (T.S.); raluca.stoian@uniklinik-freiburg.de (R.S.); constantinos.zamboglou@uniklinik-freiburg.de (C.Z.); eleni.gkika@uniklinik-freiburg.de (E.G.); anca.grosu@uniklinik-freiburg.de (A.-L.G.); 2 German Cancer Consortium (DKTK) Partner Site Freiburg, German Cancer Research Center (DKFZ), Neuenheimer Feld 280, 69120 Heidelberg, Germany; 3 Department of Molecular Radiation Oncology, German Cancer Research Center (DKFZ), Neuenheimer Feld 280, 69120 Heidelberg, Germany; 4 Department of Otorhinolaryngology, University of Freiburg—Medical Center, Killianstr. 5, 79106 Freiburg, Germany; andreas.knopf@uniklinik-freiburg.de

**Keywords:** head-and-neck cancer, head-and-neck squamous cell carcinoma, radiotherapy, chemotherapy, elderly patients, albumin, C-reactive protein

## Abstract

The purpose of this study was to evaluate the value of routine blood markers regarding their predictive potential for treatment outcomes of elderly head-and-neck squamous cell carcinoma (HNSCC) patients. In total, 246 elderly HNSCC patients (≥65 years) undergoing (chemo)radiotherapy from 2010 to 2018 were analyzed for treatment outcomes, depending on their hemoglobin, glomerular filtration rate (GFR), C-reactive protein (CRP) and albumin values, representing anemia, kidney function, inflammation and nutrition status, respectively. Local/locoregional control, progression-free and overall survival (OS) were calculated using the Kaplan–Meier method. Cox analyses were performed to examine the influence of blood parameters on oncological outcomes. In the univariate Cox regression analysis, hemoglobin ≤ 12 g/dL (HR = 1.536, *p* < 0.05), a GFR ≤ 60 mL/min/1.73 m^2^ (HR = 1.537, *p* < 0.05), a CRP concentration > 5 mg/L (HR = 1.991, *p* < 0.001) and albumin levels ≤ 4.2 g/dL (HR = 2.916, *p* < 0.001) were significant risk factors for OS. In the multivariate analysis including clinical risk factors, only performance status (HR = 2.460, *p* < 0.05) and baseline albumin (HR = 2.305, *p* < 0.05) remained significant prognosticators. Additionally, baseline anemia correlated with the prevalence of higher-grade chronic toxicities. We could show for the first time that laboratory parameters for anemia (and at least partly, tumor oxygenation), decreased renal function, inflammation and reduced nutrition status are associated with impaired survival in elderly HNSCC patients undergoing (chemo)radiotherapy.

## 1. Introduction

With more than 650,000 new diagnoses and 300,000 deaths per year, head-and-neck squamous cell carcinoma (HNSCC) constitutes a very common malignancy with both high morbidity and mortality [1]. Although the prevalence of HPV-related HNSCC, which typically affects younger patients, is increasing, the average age of HNSCC patients at the time of diagnosis ranges between 60 and 70, and one in four HNSCC patients are older than 70 years [2,3]. Due to demographic changes, the prevalence of elderly cancer patients will increase dramatically over the next decades [4]. In most clinical trials regarding radiotherapy for HNSCC, elderly patients were either underrepresented or completely excluded, which is why clinical decision making for this patient cohort is challenging [5,6,7,8]. The clinical profile of elderly HNSCC patients is different to younger HNSCCs patients, and elderly HNSCC cohorts comprise a higher percentage of female patients and a lower presence of alcohol or tobacco abuse [9]. Chemotherapy, as well as the epidermal growth factor receptor (EGFR) antibody cetuximab, have demonstrated less efficacy in elderly HNSCC patients [10,11,12]. Similarly, altered fractionation regimens such as hyperfractionation have shown less benefit in comparison with younger HNSCC patients [13]. Therefore, there is a strong need for identifying elderly patients that may not benefit from these standard therapies and may require treatment adaption.

Over recent decades, a large number of novel blood biomarkers for HNSCC, such as miRNA patterns, were studied; however, none of these novel blood markers are currently recommended for routine clinical use [14,15]. Several blood markers are routinely assessed before and during routine clinical treatments and represent oxygenation status (e.g., hemoglobin), kidney function (e.g., glomerular filtration rate [GFR] and creatinine), systemic inflammation (e.g., C-reactive protein [CRP] and leukocytes), nutrition status (e.g., albumin) and tumor burden (e.g., lactate dehydrogenase [LDH]). These blood tests may, therefore, provide useful surrogate parameters. However, whether these routine biomarkers are able to predict survival and treatment-related toxicity rates after radiotherapy or chemoradiation for elderly HNSCC patients is unknown.

We examined the role of these surrogate laboratory parameters regarding oncological outcomes and treatment-related toxicities in a large single-center study consisting of 246 geriatric HNSCC patients receiving radiotherapy or chemoradiation.

## 2. Results

### 2.1. Patient Characteristics

A total of 246 patients with histologically confirmed HNSCC who received radiotherapy or chemoradiation between 2010 and 2018 in our department were included in this analysis. The median age of our cohort amounted to 72 years (65–96 years) with the majority exhibiting an age between 65 and 74 years (*n* = 153, 62.2%). Similar to other head-and-neck cancer studies, most patients were male (*n* = 170, 69.1%) and smokers (*n* = 142, 57.7%) [16,17]. More than half of our cohort exhibited a Karnofsky performance status of 90% (*n* = 108, 43.9%) or 100% (*n* = 28, 11.4%), and only 18 patients (7.3%) were found to have a Karnofsky performance status of 60% or less. One hundred and forty-seven patients (59.8%) received concomitant systemic treatment, mostly with platinum-based regimens. Detailed patient and treatment characteristics were described previously and can be found in Table 1 and Table S1 [18]. Fisher’s exact tests were carried out to reveal potential differences between the radiotherapy and chemoradiotherapy group. Patients in the chemoradiotherapy group were significantly younger than in the radiotherapy cohort (71 years versus 77 years in median, *p* < 0.001, unpaired *t*-test) and exhibited more frequently more advanced T and N stages (*p* < 0.001, Fisher’s exact tests).

### 2.2. Dynamics of Routine Biomarkers during Therapy

**Figure 1:**
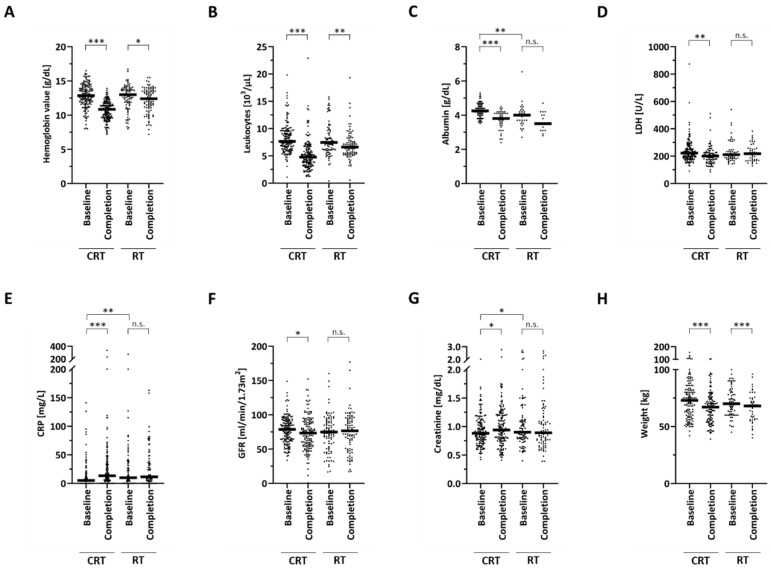
Scatter dot plots visualizing the distribution of several blood parameters, namely hemoglobin (**A**), leukocyte count (**B**), albumin (**C**), LDH (**D**), CRP (**E**), GFR (**F**), creatinine (**G**), and body weight (**H**) during the course of chemoradiation (CRT) or radiotherapy (RT). Each dot plot represents a parameter of one patient, and the horizontal line shows the median sample value. Two-sided unpaired *t*-tests were carried out to test for differences between the baseline values in the chemoradiation and radiotherapy group, while two-sided paired *t*-tests were conducted in order to detect differences between the baseline and completion groups. * *p* < 0.05, ** *p* < 0.01, *** *p* < 0.001, n.s. = not significant. Number of available values at baseline was *n* = 235 (hemoglobin), *n* = 234 (leukocytes), *n* = 129 (albumin), *n* = 199 (LDH) *n* = 145 (CRP), *n* = 234 (GFR), *n* = 234 (creatinine) and *n* = 193 (body weight).

While the hematological parameters hemoglobin and leucocyte counts did not differ between the chemoradiation and radiotherapy group at the start of treatment, patients in the radiotherapy group had significantly lower baseline albumin levels (*p* < 0.01) and higher baseline CRP values (*p* < 0.01) compared to patients treated with chemoradiation (Figure 1).

The median baseline creatinine concentration amounted to 0.92 mg/dL in the chemoradiation group, which was significantly lower than in the radiotherapy group with 1.04 mg/dL (*p* < 0.05); however, the GFR was comparable between both groups at the beginning of treatment (78 mL/min/1.73 m^2^ in the chemoradiation group versus 73.82 mL/min/1.73 m^2^ in the radiotherapy group, *p* = 0.183).

Hemoglobin concentration and leukocyte counts were observed to significantly decrease during both radiotherapy and chemoradiation (Figure 1A,B, Figure S1). However, chemoradiation led to a stronger decrease in these two parameters than radiotherapy alone. While the median hemoglobin level dropped from 12.9 to 10.9 g/dL in the chemoradiation cohort (*p* < 0.001, paired *t*-test), there was only a minor drop from 13.0 to 12.4 g/dL in the radiotherapy group (*p* < 0.05). Similarly, chemoradiation resulted in a decrease in the leukocyte levels from 7.7 × 10^3^/µL to 4.8 × 10^3^/µL (*p* < 0.001), while radiation led to a minor fall from 7.5 × 10^3^/µL to 6.6 × 10^3^/µL (*p* < 0.01). The albumin concentration was observed to significantly drop in chemoradiation, but not in the radiotherapy cohort (*p* < 0.001 for the chemoradiation group, *p* = 0.092 in the radiotherapy group) (Figure 1C). The baseline LDH concentration varied strongly among HNSCC patients with 91 U/L as the lowest and 874 U/L as the highest value. Patients undergoing chemoradiation exhibited a relatively small but significant decrease in their LDH levels (*p* < 0.01), which was not observed in patients receiving radiotherapy without concomitant chemotherapy (*p* = 0.327) (Figure 1D). Interestingly, the CRP value was found to significantly increase only in the chemoradiation but not in the radiotherapy group (Figure 1E). The median CRP value amounted to 5.0 mg/L at the beginning of chemoradiation and increased to 13.3 mg/L at the end of chemoradiation (*p* < 0.001). In contrast, the CRP value was found largely unaffected in the radiotherapy group, with a median CRP concentration of 10 and 11.5 mg/L at the start and end of treatment, respectively (*p* = 0.972). While the kidney function quantified by the GFR and creatinine concentration slightly worsened in patients treated with chemoradiation (*p* < 0.05), it remained stable in patients receiving radiotherapy alone (*p* = 0.746 regarding creatinine) (Figure 1F, Figure 1G). The patients’ body weight was significantly reduced during treatment both in the chemoradiation (73 to 67 kg; *p* < 0.001) and radiotherapy group (70 to 68 kg; *p* < 0.001) (Figure 1H). In order to reveal an association between systemic inflammation status quantified by the CRP concentration and the nutritional status quantified by the albumin concentration, we carried out bivariate correlation analyses between both of these parameters and detected a weak but significant inverse correlation (Pearson’s r = −0.276, *p* < 0.01).

### 2.3. Treatment Outcome in Dependence of Baseline Blood Parameters

**Figure 2:**
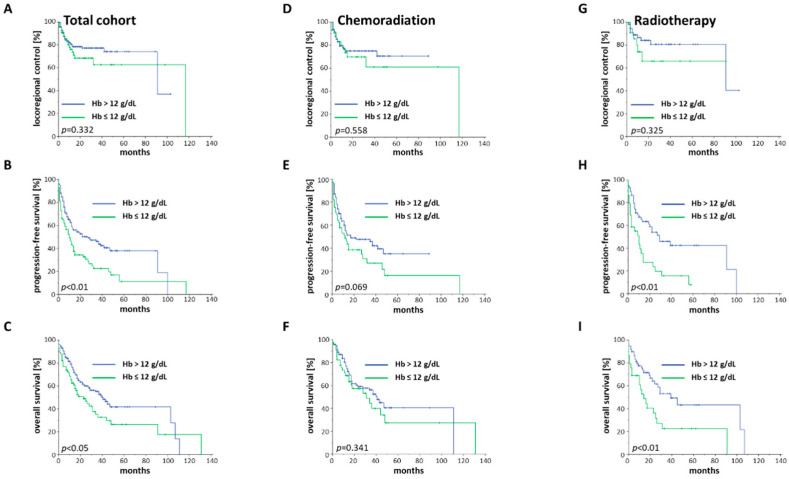
Kaplan–Meier curves showing LRC, PFS and OS of elderly HNSCC patients in dependence of the baseline hemoglobin (Hb) value. (**A**–**C**) Kaplan–Meier curves for the entire cohort with available Hb values (*n* = 235). (**D**–**F**) Kaplan–Meier curves consisting of all patients who received chemoradiation and exhibited available Hb values (*n* = 147). (**G**–**I**) Kaplan–Meier curves showing all patients who were treated with radiotherapy and had available Hb levels (*n* = 88). *p*-values of log-rank tests are shown.

The two-year overall survival (OS), progression-free survival (PFS) and local/locoregional control (LRC) of our elderly HNSCC cohort ranged at 56.9%, 44.9% and 75.5%, respectively (Figure S2) [18]. Baseline anemia, defined as a hemoglobin level below 12 g/dL, resulted in significantly impaired PFS (*p* < 0.01, log-rank test) and OS (HR = 1.536, 95% CI 1.058–2.231, *p* < 0.05), while LRC was not influenced (*p* = 0.332) (Figure 2A–C, Table 2). Fisher’s exact tests were carried out to assess whether patients with baseline anemia exhibited more advanced tumor stages; anemic patients demonstrated significantly more locally advanced tumors than non-anemic patients (*p* = 0.011), but there was no difference regarding the nodal stages between both groups (*p* = 0.913).

Interestingly, the negative prognostic influence of baseline anemia on PFS and OS was not observed in the chemoradiation group (*p* = 0.069 for PFS, *p* = 0.341 for OS) (Figure 2D–F). In contrast, baseline anemia was shown to be a highly significant prognosticator in the radiotherapy group. While elderly HNSCC patients with hemoglobin concentrations exceeding 12 g/dL exhibited a median OS of 39 months, anemic patients only survived for a median of 14 months (*p* < 0.01) (Figure 2H,I). However, this effect was not driven by the inferior LRC rates of anemic patients (*p* = 0.325) (Figure 2G). In order to examine whether a peri-therapeutic decrease in hemoglobin, which is likely mainly caused by concomitant chemotherapy, may be associated with reduced survival rates, we assessed the LRC, PFS and OS rates in dependence of the peri-therapeutic hemoglobin changes (Figure S3). A hemoglobin drop did not result in reduced LRC (*p* = 0.547), PFS (*p* = 0.374) or OS rates (*p* = 0.162), and these findings were consistent for the chemoradiation and radiotherapy groups.

**Figure 3:**
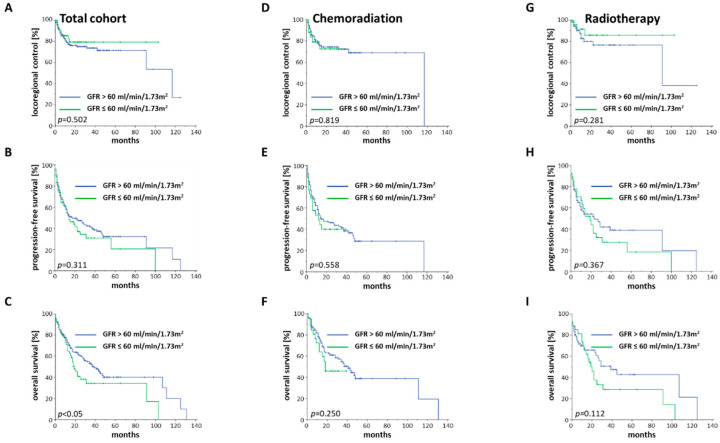
LRC, PFS and OS of elderly HNSCC patients stratified by the baseline GFR value. (**A**–**C**) Kaplan–Meier curves for the entire cohort with existing GFR values (*n* = 234). (**D**–**F**) Kaplan–Meier curves consisting of all patients who received chemoradiation and had accessible GFR values (*n* = 146). (**G**–**I**) Kaplan–Meier curves showing all patients who were treated with radiotherapy and had available GFR levels (*n* = 88). Log-rank tests were performed for comparisons.

Reduced renal function, defined as a GFR below 60 mg/mL/1.73 m^2^, has been described as a negative prognosticator for other malignancies and was, therefore, analyzed in our elderly HNSCC cohort. Impaired glomerular function prior to treatment was found to significantly reduce OS (HR = 1.537, 95% CI 1.024–2.308, *p* < 0.05), whereas PFS and LRC were observed comparable between both groups (Figure 3A–C).

As patients with pre-therapeutically impaired kidney function often did not receive concomitant chemoradiation, only a few patients could be compared for the chemoradiation cohort, thereby impairing statistical analyses (Figure 3D–F). Elderly HNSCC patients with reduced kidney function who were treated with radiotherapy alone showed a trend towards lower OS, although statistical significance was not reached (*p* = 0.112). Neither PFS (*p* = 0.367) nor LRC (*p* = 0.281) were found to vary, depending on the baseline GFR (Figure 3G–I).

**Figure 4:**
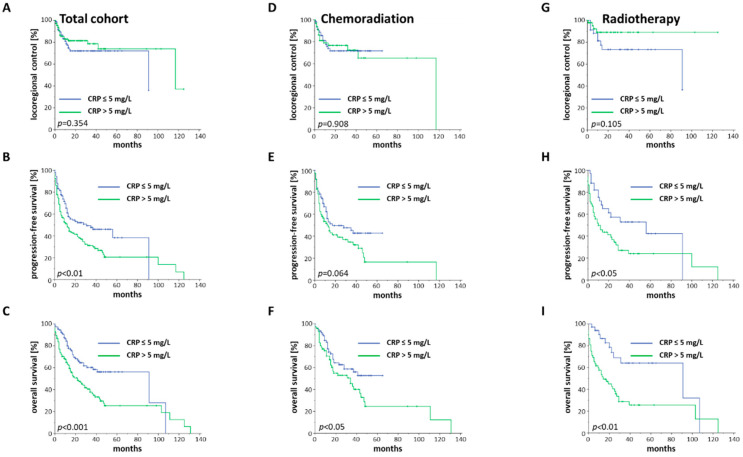
LRC, PFS and OS of elderly HNSCC patients stratified by the baseline CRP concentration. (**A**–**C**) Kaplan–Meier curves for the complete cohort with accessible CRP values (*n* = 231). (**D**–**F**) Kaplan–Meier curves consisting of patients who were treated with chemoradiation and had accessible CRP values (*n* = 145). (**G**–**I**) Kaplan–Meier curves showing all patients who received radiotherapy and had available CRP levels (*n* = 86). *p*-values of log-rank tests are shown.

A cut-off of 5 mg/L for CRP was chosen, based on the given normal range of the acute phase protein. Using this cut-off, both the PFS (*p* < 0.01) and OS (HR = 1.991, 95% CI 1.356–2.923, *p* < 0.001) were significantly reduced for baseline CRP exceeding 5 mg/L with no differences in terms of the LRC rates (*p* = 0.354) (Figure 4A–C).

While the baseline CRP concentration did not affect LRC after chemoradiation (*p* = 0.908) or radiation (*p* = 0.105), OS was lower for patients with increased CRP after chemoradiation (*p* < 0.05) as well as after radiotherapy (*p* < 0.01). In the radiotherapy cohort, patients treated with radiotherapy and CRP within the normal range exhibited a median OS of 91 months, which was more than six times higher than patients with elevated CRP at baseline (median OS of 14 months). PFS rates were only significantly influenced by increased CRP serum levels in the radiotherapy group (*p* < 0.05) but not in the chemoradiation (*p* = 0.064) group (Figure 4D–I).

**Figure 5:**
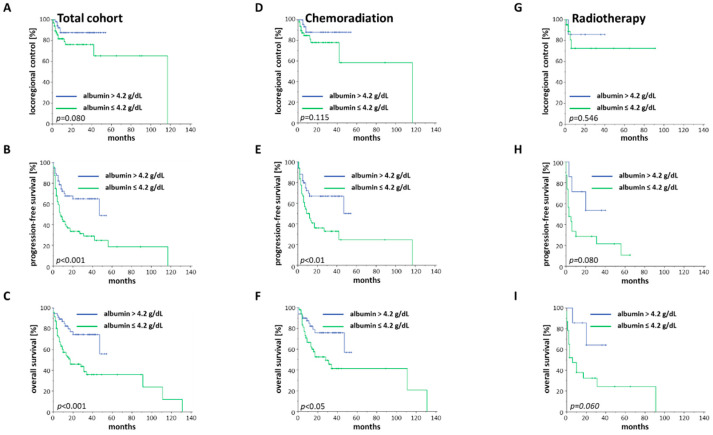
LRC, PFS and OS of elderly HNSCC patients in dependence of the baseline albumin serum level (**A**–**C**) Kaplan–Meier curves for the entire cohort with existing albumin serum values (*n* = 129). (**D**–**F**) Kaplan–Meier curves of all patients undergoing chemoradiation who had available albumin values (*n* = 99). (**G**–**I**) Kaplan–Meier curves showing all patients who were treated with radiotherapy and had available albumin values (*n* = 30). *p*-values of log-rank tests are shown.

For albumin as a potential surrogate for the patients’ nutritional status, we used the median value of 4.2 g/dL as a cut-off. Survival differences regarding PFS and OS were considerably more pronounced using baseline albumin concentration compared to any of the other tested lab results (Figure 5A–C).

While the median OS for patients with an baseline albumin concentration above 4.2 g/dL was not reached, patients with albumin levels below this cut-off value exhibited a median OS of 17 months (HR = 2.916, 95% CI 1.561–5.445, *p* < 0.001). There was even a non-significant trend towards improved LRC for patients with albumin levels higher than 4.2 g/dL (*p* = 0.080). Using a cut-off of 3.5 g/dL, which is the given lower limit of the physiological range, elderly HNSCC patients with hypoalbuminemia had a median OS of only three months, being 60-times lower than the median OS of patients without hypoalbuminemia (*p* < 0.001). Patients undergoing chemoradiation had superior PFS (*p* < 0.01) and OS (*p* < 0.05), if the baseline albumin value was more than 4.2 g/dL (Figure 5D–F). In the radiotherapy group, lower albumin levels resulted in a trend towards reduced PFS (*p* = 0.080) and OS (*p* = 0.060), with no differences in terms of the LRC (*p* = 0.546) (Figure 5G–I).

The LDH serum concentration has been shown to be a prognostic marker for several malignancies and represents a measure for tumor burden, tumor necrosis and tumor glycolysis [19,20,21]. Increased pre-therapeutic LDH serum levels, defined as a LDH concentration above 250 U/L, did not result in reduced LRC (*p* = 0.179), PFS (*p* = 0.439) or OS rates (*p* = 0.651) for the complete cohort (Figure S4). However, elderly HNSCC patients who received radiotherapy without concomitant chemotherapy had improved OS rates, if baseline LDH serum levels were below 250 U/L (*p* < 0.05).

Univariate Cox analyses revealed a reduced performance status, specified as a Karnofsky index lower than 80% (HR = 2.803, 95% CI 1.817–4.324, *p* < 0.001), an age above 75 years (HR = 1.584, 95% CI 1.101–2.280, *p* < 0.05) and a positive smoking status (HR = 1.731, 95% CI 1.045–2.868, *p* < 0.05) as significant clinical prognostic factors for impaired OS. Interestingly, neither the T (HR = 1.267, 95% CI 0.863–1.861, *p* = 0.227) nor the N status (HR = 1.099, 95% CI 0.746–1.617, *p* = 0.633) influenced the OS of elderly HNSCC patients. We included both the significant clinical parameters and blood values in a multivariate analysis and performed a backward stepwise Cox regression analysis with likelihood ratio tests. In this analysis, only the performance status (HR = 2.460; 95% CI 1.227–4.930; *p* < 0.05) and the baseline albumin serum level (HR = 2.305; 95% CI 1.060–5.011; *p* < 0.05) remained significant prognosticators for OS. Spearman’s correlation analyses of both significant parameters in the multivariate analysis demonstrated a significant correlation between albumin and the Karnofsky performance status (Spearman’s ρ = 0.290, *p* < 0.01). In the multivariate analysis, increased age was found to be a borderline-significant risk factor for reduced OS (HR = 1.918; 95% CI 0.992–3.708; *p* = 0.053), whereas smoking status and the blood parameters hemoglobin, GFR and CRP had no significant impact on the survival rates.

In order to detect potential differences between the different age groups, we separately carried out Cox regression analyses for patients between 65 and 74 years (“young olds”, *n* = 153, 62.2%) and patients aged 75 years and older (“older olds and oldest olds”, *n* = 93, 37.8%), according to the definition of geriatric subgroups (Table 3) [9].

For the so called younger olds, a reduced performance status (HR = 2.108, 95% CI 1.090–4.075, *p* < 0.05), a positive smoking status (HR = 2.733, 95% CI 1.168–6.393, *p* < 0.05) and an increased CRP (HR = 1.782, 95% CI 1.073–2.960, *p* < 0.05) were negative prognosticators regarding OS. For patients aged 75 years and older, the performance status remained a highly significant prognostic parameter (HR = 3.218, 95% CI 1.748–5.926, *p* < 0.001). Interestingly, the laboratory parameters for anemia (hemoglobin ≤ 12 g/dL: HR = 2.153, 95% CI 1.220–3.797, *p* < 0.01), systemic inflammation (CRP > 5 mg/L: HR = 2.317, 95% CI 1.255–4.280, *p* < 0.01) and impaired nutrition status (albumin ≤ 4.2 g/dL: HR = 3.224, 95% CI 1.227–8.477, *p* < 0.05) were found to correspond to reduced OS rates in this subgroup. Due to the limited patient number in the age subgroups, we did not perform multivariate analyses.

### 2.4. Routine Biomarkers as Potential Prognosticators for Treatment-Associated Toxicities

**Figure 6:**
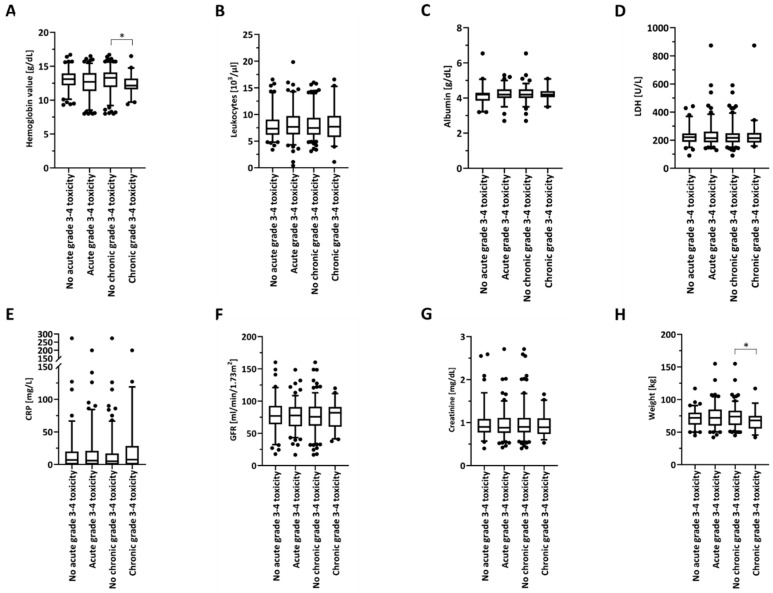
Box-plot diagram showing various baseline blood parameters, namely hemoglobin (**A**), leukocyte count (**B**), albumin (**C**), LDH (**D**), CRP (**E**), GFR (**F**), creatinine (**G**), and body weight (**H**) in dependence of acute or chronic CTCAE grade 3–4 toxicities. The box extends from the first to the third quartile, while the horizontal line shows the median sample value. The whiskers range from the 5th percentile to the 95th percentile, and all other points are plotted as outliers. Two-sided unpaired *t*-tests were performed for comparisons. * *p* < 0.05.

Moderate to severe acute toxicities were found to be relatively high in our elderly patient cohort, with 56.1% of patients (*n* = 138) suffering from at least one acute CTCAE grade ≥3 toxicity (Table S2). The most common treatment-related grade ≥3 toxicities were dysphagia, cytopenia and mucositis with 80 (32.5%), 69 (28.0%) and 46 (18.7%) affected patients, respectively (Table S3). A total of 45 patients (19.9%) developed at least one chronic grade ≥3 toxicity, most frequently dysphagia, jaw/dental injuries and pain with 31 (13.7%), 10 (4.4%) and 4 (1.8%) events, respectively. While higher-grade acute toxicities can reduce treatment compliance and lead to unplanned radiotherapy interruptions, thereby decreasing oncological outcomes, higher-grade chronic toxicities can severely impair the quality of life of surviving HNSCC patients. Therefore, it is crucial to identify risk factors both for higher-grade acute and chronic toxicities. Interestingly, baseline anemia was shown to be more prevalent in patients exhibiting higher-grade chronic toxicities: 31.8% of the patients with chronic grade 3 toxicities exhibited baseline hemoglobin values below 12 g/dL, which was more than twice as much (14.7%) than in patients not suffering from higher-grade chronic toxicities (*p* < 0.01, χ^2^-test) (Figure 6A, Table 4).

Dysphagia (*n* = 14) was the most common chronic grade 3 toxicity in the anemic patients, followed by jaw and dental injuries (*n* = 2). Other parameters, such as baseline leukocytes, albumin, LDH, CRP, GFR and creatinine levels were comparable between patients without and with higher-grade toxicities (Figure 6B–G). Patients with a body weight lower than the median of 72 kg suffered more frequently from higher-grade chronic toxicities (25.8% versus 14.1%, *p* < 0.05) (Figure 6H, Table 4). Similar to the anemic patients, dysphagia (*n* = 12) was the most frequent chronic grade 3 toxicity in patients with a weight below 72 kg, followed by kidney failure (*n* = 3).

## 3. Discussion

In this large analysis, we have shown the prognostic strength of the routine blood markers hemoglobin, GFR, CRP and albumin as surrogate parameters for anemia (thereby also, at least partly and indirectly, tumor oxygenation), renal function, systemic inflammation and nutrition status of elderly HNSCC patients undergoing (chemo)radiotherapy. While tumor staging did not predict the survival of elderly HNSCC patients after (chemo)radiotherapy, established and easily obtainable blood markers were significantly associated with the oncological outcomes in this distinct and vulnerable patient cohort. Indeed, the baseline albumin serum level remained the only significant risk factor together with the performance status as an established prognosticator in the multivariate analysis, showing the importance of this blood marker as a surrogate for the nutrition status.

Although the role of albumin as a prognosticator for HNSCC has been suggested previously, the role of albumin in elderly HNSCC patients is unknown so far, and it may be even more important for the more frail geriatric cancer population [22,23,24]. Albumin is an indicator of a patient’s nutritional status, and hypoalbuminemia is considered as a potential biomarker for sarcopenia. Chargi and colleagues have demonstrated in a retrospective study consisting of 85 elderly HNSCC patients that sarcopenia, defined as combination of low skeletal muscle mass and low muscle function, was present in almost half of their elderly HNSCC cohort and significantly deteriorated the survival of these patients [25]. Malnutrition is considered as a major cause for sarcopenia, and the prevalence of malnutrition in HNSCC patients is high, with up to 88% of HNSCC patients suffering from malnutrition at the end of radiotherapy [26,27]. Malnutrition of HNSCC patients is a multifactorial process, in which dysphagia, inappetence, decreased physical activity, often-existing ethanol consumption and hypercatabolism due to systemic inflammation play an important role [28]. Although the correlation was moderate, CRP and albumin inversely correlated with each other in our study, suggesting an association of these parameters in elderly HNSCC patients. Albumin acts as a negative acute-phase protein, suggesting that reduced albumin may indicate increased systemic inflammation, which is known to negatively affect oncological outcomes [29]. Unfortunately, malnutrition is not routinely assessed in a standardized manner for HNSCC patients in many institutions, although several guidelines recommend routine assessment of the patient’s nutrition status prior to starting cancer treatment, especially for elderly patients [30,31]. Whether routine nutritional interventions such as enteral or parenteral substitution during (chemo)radiotherapy may improve the outcomes of elderly HNSCC patients with low baseline albumin levels remains to be addressed.

The negative impact of anemia regarding the outcomes after radiotherapy has been described for several malignancies including cervical carcinoma and HNSCC [32,33]. It has been shown that severe anemia correlates with tumor-associated hypoxia in HNSCC, thereby increasing the radiation resistance of the tumor tissue, and thus, impairing the antitumor efficacy of radiotherapy [34]. Intratumoral hypoxia during radiotherapy for HNSCC has been demonstrated to serve as a prognostic indicator for a higher risk of locoregional relapse [35,36]. Large studies have shown that attempts to correct anemia by erythropoietin did increase the hemoglobin value but did not lead to improved oncological outcomes in HNSCC patients undergoing radiotherapy [37,38]. Similarly, the DAHANCA 5 and 7 trials demonstrated that blood transfusions for HNSCC patients with low hemoglobin (females < 13 g/dL; males < 14.5 g/dL) only corrected the measured hemoglobin levels but did not improve the oncological outcomes after chemoradiation [39]. As we did not observe differences in terms of the LRC, additional non-cancer effects may contribute to the inferior results for anemic patients. The prevalence of anemia is increasing with advancing age, indicated by the fact that more than 20% of people aged 85 years or older exhibit anemia [40,41], and anemia is considered as an independent risk factor for hospitalization, morbidity (e.g., cognitive decline) and mortality in elderly people [40,42]. The risk of cardiovascular events is significantly higher in anemic compared to non-anemic people, which may explain the results observed in our study [43].

The prognostic role of CRP serum levels has been shown for a variety of different malignancies such as glioblastoma, breast cancer and ovarian cancer [44,45,46]. Additionally, baseline CRP levels were recently found to predict prognosis of HNSCC patients; however, its role, especially for elderly HNSCC patients undergoing (chemo)radiotherapy, is unknown [17,47,48]. There are different possible explanations for the inverse association between CRP levels and survival rates. Increased CRP levels may indicate a more aggressive tumor and pronounced inflammation inside the tumor microenvironment [49]. Interleukin-6 (Il-6) is an established risk factor for inferior oncological outcomes of HNSCC patients, and Il-6 has been demonstrated to be associated with HNSCC progression, angiogenesis and metastasis [50]. As Il-6 acts as an inducer of the CRP gene, CRP may only be a variable surrogate for higher Il-6 levels. In addition, an elevated CRP serum concentration is known to increase the risk of cardiovascular events, which is a common cause of death in the elderly HNSCC population [51]. This hypothesis is supported by the finding that the LRC was found unaffected by higher CRP levels and the decreased OS may therefore be due to non-cancer-associated events.

The prognosis of elderly HNSCC patients is also determined by treatment-related toxicities. Especially treatment-related late toxicities may severely reduce the quality of life for a long time after curative treatment, which is why prognostic parameters are needed to identify patients at an increased risk of higher-grade toxicities; this seems even more crucial for elderly patients for whom the quality of life is commonly a key factor for treatment decisions [52]. Correlative analyses between routine biological parameters and treatment-related toxicities revealed anemia and nutrition status as prognostic factors. Elderly HNSCC patients with baseline anemia exhibited a more than two-fold higher risk of severe chronic toxicities compared to non-anemic patients. Similarly, patients with a body weight below the median value of 72 kg had an almost two times higher risk of developing grade 3 toxicities. Pre-therapeutic weight loss may be a surrogate parameter for pre-existing dysphagia, which could contribute to the higher prevalence of dysphagia after treatment in patients who weigh less than the median body weight. A more causal explanation is that a sufficient nutritional status and plasma protein levels are necessary for an adequate repair of normal tissue injuries caused by ionizing radiation, suggesting that patients who are underweight do not have enough protein reserves to repair irradiation-induced normal tissue damages. Since the exact interplay between these parameters and chronic toxicities is unknown, further studies are warranted to examine a potential prognostic role of baseline anemia and underweight for high-grade chronic toxicities such as dysphagia.

Routine blood markers for risk prediction of elderly HNSCC patients have several advantages. In contrast to geriatric or comorbidity burden assessments, these parameters are easily available without specific assessments and are commonly analyzed as part of the pre-treatment routine. As the evaluation of performance status has high inter-observer variability, laboratory results of these blood parameters may be advantageous due to higher reliability. Additionally, longitudinal measurements are easy to obtain and may further help to refine the prognostic validity.

The relatively poor survival rates in comparison to the respective LRC may be a sign for the advanced age and vulnerability of this vulnerable patient cohort, which may also explain the fact that the investigated blood parameters were found to influence survival but not the LRC in our analysis. Many elderly HNSCC patients may not have died as a direct consequence of cancer progression but due to their comorbidities. For instance, a large cancer database analysis showed that cancer progression was the cause of death for only about half of elderly HNSCC patients [53].

Albeit being a comprehensive investigation based on a large cohort of elderly HNSCC patients, our analysis has some limitations. Due to the retrospective nature of the study, not all laboratory results were available for all patients. Additionally, HPV as a well-known prognostic biomarker for oropharyngeal carcinomas was assessed only for 83 patients due to the time period of the study and was, therefore, not included in the multivariate analysis. Furthermore, we did not routinely assess the alcohol intake of elderly HNSCC patients, although alcohol consumption is known to influence nutritional status in many ways. The high energy content of alcohol may help to reach the recommended caloric intake while receiving insufficient amounts of many essential nutrients including proteins. Additionally, alcohol can impair sufficient absorption and digestion of these essential nutrients [54,55]. As we did not validate our observations in an independent cohort, our data should be interpreted as hypothesis-generating. Therefore, further studies are needed in order to confirm the prognostic role of these markers for elderly HNSCC patients undergoing (chemo)radiotherapy.

## 4. Materials and Methods

### 4.1. Patients and Treatment

All HNSCC patients aged 65 years or older who were treated with radiotherapy or chemoradiation between 2010 and 2018 at the Department of Radiation Oncology, University of Freiburg Medical Center, were included in this retrospective analysis. Demographic characteristics and clinical data were collected using electronic patient records. Pathological data were obtained from pathology reports, and the analyzed blood values were extracted from the laboratory results. Smoking was defined as tobacco consumption of more than 10 pack years. Due to the time period of the study, staging of HNSCCs was performed according to the 7th Edition of the UICC TNM classification. We did not translate the tumor stages according to the 8th Edition of the UICC TNM classification, as the HPV status was not available for all patients with oropharyngeal carcinoma.

Treatment decisions were based on the recommendations of the multidisciplinary tumor board. For treatment planning and application, HNSCC patients were immobilized with a thermoplastic head-and-neck mask. Radiotherapy planning was performed using Oncentra MasterPlan^®^ (Nucletron BV, Veenendaal, The Netherlands) and EclipseTM planning software (Varian Medical Systems). Three-dimensional conformal radiotherapy (3DRT) as well as intensity-modulated radiotherapy (IMRT) were used for treatment. Radiotherapy was administered in 2 Gy single fractions to a total dose of 70 Gy for definitive and 60–66 Gy for adjuvant treatments.

If there were no medical contraindications, concurrent chemotherapy was administered for all HNSCC patients receiving definitive radiotherapy treatment. In the adjuvant setting, concomitant chemoradiation was applied in the case of incomplete resection or extranodal extension (ENE).

Ethical approval for this study was obtained from the Independent Ethics Committee of the University of Freiburg (reference no. 551/18).

### 4.2. Survival and Toxicity

During the first year, patients presented 3-monthly for follow-up consultations, and follow-up was scheduled in 6-monthly intervals between years 2 and 5. Follow-up consultations contained a physical examination and cross-sectional imaging of the head-and-neck region using CT or MR imaging. The record sections of the federal state authorities of Baden-Württemberg were contacted in order to acquire missing survival data. OS was calculated as the time from treatment completion to death from any cause, and PFS was defined as the interval between treatment completion and disease progression at any site or death of any cause. LRC was defined as the time from treatment completion to the absence of any progression of the primary tumor or cervical lymph node metastases. Patients lost to follow-up were censored. Acute and chronic treatment-related toxicities were classified based on the Common Terminology Criteria of Adverse Effects version 5.0. Acute toxicities were defined as adverse reactions during radiotherapy and within the first 90 days after completion of radiotherapy; chronic toxicities were counted if occurring later than 90 days.

### 4.3. Blood Value and Body Weight Analyses

Analyzed blood results of HNSCC patients included the hematological parameters hemoglobin and leukocyte count, kidney function (GFR and creatinine concentration), inflammation parameters such as CRP, nutritional parameters such as albumin and the tissue turnover and tumor burden parameter LDH. These blood parameters usually were checked weekly during radiotherapy. HNSCC patients undergoing chemoradiation were hospitalized during chemotherapy application and received blood sampling more frequently if necessary. Patients’ blood results and body weight were retrospectively assessed from the patient charts. Baseline was defined as either the day of treatment initiation or up to 5 days before, while completion was specified as the last day or up to 5 days prior to the end of radiotherapy or chemoradiation.

### 4.4. Statistical Analysis

LRC, PFS and OS were calculated according to the Kaplan–Meier method, and log-rank tests were used for statistical comparisons between individual Kaplan–Meier curves. The median follow-up of our study population was determined using the reverse Kaplan–Meier method. Uni- and multivariate Cox proportional hazards model analyses were performed in order to examine the impact of clinicopathological characteristics and blood results on patient survival. All parameters that were shown to be significant risk factors in the univariate analysis were included in the multivariate analysis, in which a backward stepwise Cox regression analysis with likelihood ratio tests was conducted. Fisher’s exact tests were used to examine whether the chemoradiotherapy group exhibits different patient characteristics such as sex, age, smoking, Karnofsky performance status, TNM status, grading and HPV status compared to the radiotherapy group. In addition, Fisher’s exact tests were applied to test for differences in the T and N stage between anemic and non-anemic patients. Two-sided unpaired *t*-tests were carried out to test for differences between the baseline values in the chemoradiation and radiotherapy group. Baseline and completion blood results were compared using paired *t*-tests, while potential differences in the prevalence of acute and chronic higher-grade toxicities in dependence of anemia (hemoglobin ≤ 12 g/dL), systemic inflammation (CRP > 5 mg/L), impaired nutrition status (albumin ≤ 4.2 g/dL), reduced renal function (GFR) ≤ 60 mg/mL/1.73 m^2^), tumor burden (LDH > 250 U/L) and lower body weight (≤72 kg) were tested with Pearson’s Chi-squared tests (χ^2^ tests). Pearson’s analyses between the CRP and albumin levels were conducted to obtain a correlation coefficient between both of these parameters, while Spearman’s correlations were used for comparisons between albumin and the Karnofsky status as an ordinal scale. Statistical significance was defined as *p* < 0.05 throughout the study. IBM SPSS Statistics software version 25 (IBM, Armonk, NY, USA) and GraphPad Prism software version 8 (GraphPad Software, San Diego, CA, USA) were used for statistical analyses.

## 5. Conclusions

In summary, our large dataset of elderly HNSCC patients shows the prognostic strength of the blood parameters hemoglobin, GFR, CRP and albumin, representing established features of the tumor and normal tissue biology such as oxygenation, kidney function, inflammation and nutritional status. For elderly patients, these markers seem to have more prognostic weight than established tumor characteristics, such as tumor and nodal stage. The fact that the baseline albumin concentration remains a significant prognosticator in the multivariate analysis shows the importance of this parameter for elderly HNSCC patients undergoing (chemo)radiotherapy. Whether patients with low baseline albumin levels may benefit from nutrition interventions during (chemo)radiotherapy, with regard to oncological outcomes, has to be investigated in further studies.

## Figures and Tables

**Table 1:** Patient characteristics of 246 elderly HNSCC patients undergoing (chemo)radiotherapy between 2010 and 2018 (*n* = 246). *p*-values are derived from Fisher’s exact tests in order to compare the radiotherapy (RT) with the chemoradiotherapy (CRT) group.

Parameter		Total Cohort (*n* = 246)		RT (*n* = 99)		CRT (*n* = 147)		*p*
		*n*	%	*n*	%	*n*	%	
Sex	male	170	69.1	67	67.7	103	70.1	0.779
	female	76	30.9	32	32.3	44	29.9	
Age	65–74 years	153	62.2	45	45.5	108	73.5	<0.001
	≥75 years	93	37.8	54	54.5	39	26.5	
Smoking	non-smoker	54	22.0	18	18.2	36	24.5	1.000
	smoker	142	57.7	48	48.5	94	63.9	
	missing	50	20.3	33	33.3	17	11.6	
Karnofsky	100%	28	11.4	9	9.1	19	12.9	0.109
	90%	108	43.9	38	38.4	70	47.6	
	80%	51	20.7	20	20.2	31	21.1	
	70%	23	9.3	9	9.1	14	9.5	
	60%	14	5.7	10	10.1	4	2.7	
	50%	3	1.2	2	2.0	1	0.7	
	40%	1	0.4	1	1.0	0	0.0	
	missing	18	7.3	10	10.1	8	5.4	
T stage	T1	35	14.2	23	23.2	12	8.2	<0.001
	T2	53	21.5	28	28.3	25	17.0	
	T3	64	26.0	20	20.2	44	29.9	
	T4	86	35.0	24	24.2	62	42.2	
	missing	8	3.3	4	4.0	4	2.7	
N stage	N0	82	33.3	49	49.5	33	22.4	<0.001
	N1	33	13.4	16	16.1	17	11.6	
	N2	120	48.8	32	32.3	88	59.9	
	N3	11	4.5	2	2.0	9	6.1	
M stage	M0	232	94.3	93	93.9	139	94.6	1.000
	M1	10	4.1	4	4.0	6	4.1	
	missing	4	1.6	2	2.0	2	1.4	
Grading	G1	6	2.4	5	5.1	1	0.7	0.207
	G2	157	63.8	62	62.6	95	64.6	
	G3	74	30.1	29	29.3	45	30.6	
	G4	1	0.4	0	0.0	1	0.7	
	missing	8	3.3	4	4.0	4	2.7	
HPV status	HPV-negative	49	19.9	17	17.2	32	21.8	0.643
	HPV-positive	34	13.8	10	10.1	24	16.3	
	missing	163	66.3	72	72.7	91	61.9	

**Table 2:** Univariate and multivariate analyses of clinical factors and blood parameters at baseline regarding OS in elderly HNSCC patients receiving radiotherapy or chemoradiation (*n* = 246).

Parameter	HR	CI 95%	*p*-Value
Univariate			
Karnofsky < 80/≥ 80%	2.803	1.817–4.324	<0.001
Age ≥ 75/65-74 years	1.584	1.101–2.280	0.013
Smoker/non-smoker	1.731	1.045–2.868	0.033
T3-T4/T1-T2	1.267	0.863–1.861	0.227
N1-N3/N0	1.099	0.746–1.617	0.633
Baseline GFR ≤ 60/> 60 mL/min/1.73 m^2^	1.537	1.024–2.308	0.038
Baseline Hb ≤ 12/> 12 g/dL	1.536	1.058–2.231	0.024
Baseline CRP > 5/≤ 5 mg/L	1.991	1.356–2.923	<0.001
Baseline albumin ≤ 4.2/> 4.2 g/dL	2.916	1.561–5.445	<0.001
Baseline LDH > 250/≤ 250 U/L	1.106	0.713–1.715	0.654
Baseline leukocytes < 4/4-0 × 10^3^/µL	0.703	0.173–2.858	0.623
Baseline leukocytes > 10/4-10 × 10^3^/µL	1.158	0.737–1.818	0.525
Baseline body weight ≤ 72/> 72 kg	1.079	0.712–1.637	0.719
Multivariate			
Karnofsky < 80/≥ 80%	2.460	1.227–4.930	0.011
Age ≥ 75/65-74 years	1.918	0.992–3.708	0.053
Smoker/non-smoker	1.670	0.710–3.931	0.240
Baseline GFR ≤ 60/> 60 mL/min	0.582	0.247–1.373	0.216
Baseline Hb ≤ 12/> 12 g/dL	1.433	0.686–2.995	0.339
Baseline CRP > 5/≤ 5 mg/L	1.136	0.548–2.353	0.732
Baseline albumin ≤ 4.2/> 4.2 g/dL	2.305	1.060–5.011	0.035

**Table 3:** Univariate analysis of baseline parameters regarding OS in dependence of the age group of elderly HNSCC patients (*n* = 246).

Univariate	Age 65–74 Years			Age ≥ 75 Years		
	HR	CI 95%	*p*	HR	CI 95%	*p*
Karnofsky < 80/≥ 80%	2.108	1.090–4.075	0.027	3.218	1.748–5.926	<0.001
Smoker/non-smoker	2.733	1.168–6.393	0.020	1.435	0.736–2.797	0.289
T3-T4/T1-T2	1.112	0.673–1.838	0.679	1.607	0.872–2.960	0.128
N1-N3/N0	1.616	0.911–2.866	0.101	0.815	0.468–1.420	0.470
Baseline GFR ≤ 60/> 60 mL/min	1.636	0.866–3.091	0.129	1.086	0.620–1.902	0.773
Baseline Hb ≤ 12/> 12 g/dL	1.215	0.734–2.011	0.449	2.153	1.220–3.797	0.008
Baseline CRP > 5/≤ 5 mg/L	1.782	1.073–2.960	0.026	2.317	1.255–4.280	0.007
Baseline albumin ≤ 4.2/> 4.2 g/dL	2.274	0.972–5.320	0.058	3.224	1.227–8.477	0.018
Baseline LDH > 250/≤ 250 U/L	1.470	0.853–2.535	0.166	0.765	0.351–1.666	0.500
Baseline body weight ≤ 72/> 72 kg	1.080	0.620–1.882	0.785	1.010	0.533–1.915	0.976

**Table 4:** Cross table showing the number of elderly HNSCC patients who experienced chronic grade 3–4 toxicities in dependence of the baseline hemoglobin (Hb) value and body weight. Groups were compared using χ^2^-tests.

Parameter	No Chronic Grade 3–4 Toxicities	Chronic Grade 3–4 Toxicities
Hb > 12 g/dL	128	22
Hb ≤ 12 g/dL	45	21
*p* < 0.01 (χ^2^-test)		
Body weight > 72 kg	79	13
Body weight ≤ 72 kg	69	24
*p* < 0.05 (χ^2^-test)		

## Supplementary Materials

The following are available online at https://www.mdpi.com/2072-6694/12/6/1698/s1, Figure S1: Distribution of several blood values and body weight at the beginning and at the end of (chemo)radiotherapy treatment, Figure S2: Oncological outcomes of the complete patient cohort consisting elderly HNSCC patients treated by (chemo)radiotherapy in our institution between 2010 and 2018 (*n* = 246), Figure S3: Oncological outcomes in dependence of the peri-therapeutic hemoglobin change, Figure S4: Oncological outcomes stratified by the baseline LDH concentration, Table S1: Patient characteristics consisting elderly HNSCC patients treated by (chemo)radiotherapy in our institution between 2010 and 2018 (*n* = 246), Table S2: Toxicity results after (chemo)radiotherapy of elderly patients with HNSCC according to the Common Terminology Criteria for Adverse Events (CTCAE) v5.0., Table S3: Toxicity results consisting various (chemo)radiotherapy-related adverse reactions according to the Common Terminology Criteria for Adverse Events (CTCAE) v5.0.
