# Supplementary material for: The Value of Laboratory Parameters for Anemia, Renal Function, Systemic Inflammation and Nutritional Status as Predictors for Outcome in Elderly Patients with Head-and-Neck Cancers
Source: Cancers (Basel). 2020 Jun 26;12(6):1698. doi: 10.3390/cancers12061698 (PMC7352755; doi:10.3390/cancers12061698)
Supplement: Supplementary file 1 [file cancers-12-01698-s001.pdf]

Supplementary Materials

The Value of Laboratory Parameters for Anemia, Renal Function, Systemic Inflammation and Nutritional Status as Predictors for Outcome in Elderly Patients with Head-and-Neck Cancers

Alexander Rühle, Erik Haehl, Hélène David, Tobias Kalckreuth, Tanja Sprave, Raluca Stoian, Constantinos Zamboglou, Eleni Gkika, Andreas Knopf, Anca-Ligia Grosu and Nils Henrik Nicolay

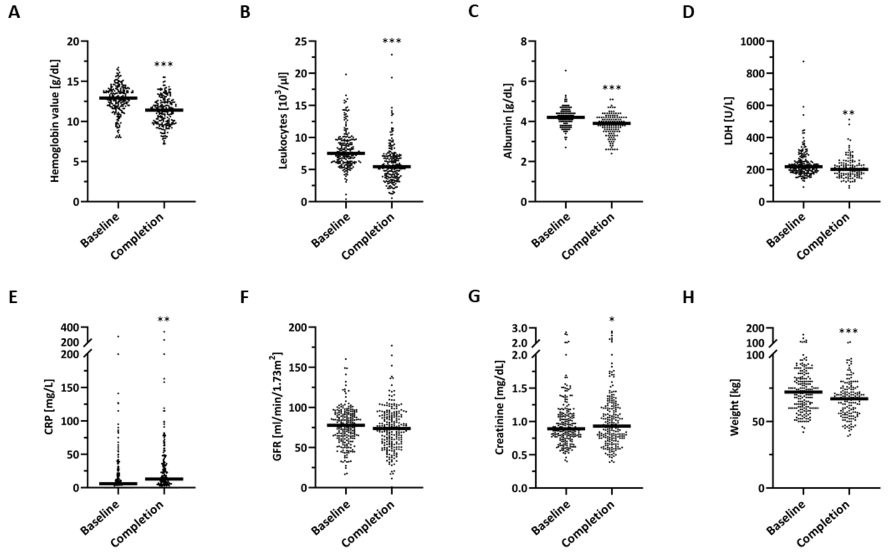

Supplementary Figure 1. Scatter dot plots demonstrating the distribution of several blood values and body weight at the beginning and at the end of (chemo)radiotherapy treatment. Groups were compared using two-sided paired t-tests. *p<0.05, **p<0.01, ***p<0.001.

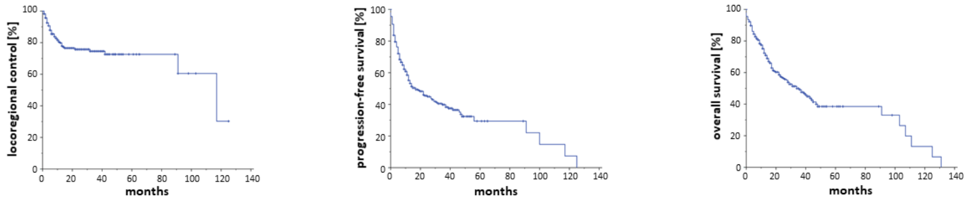

Supplementary Figure 2. LRC, PFS and OS of the complete patient cohort consisting elderly HNSCC patients treated by (chemo)radiotherapy in our institution between 2010 and 2018 (n=246).

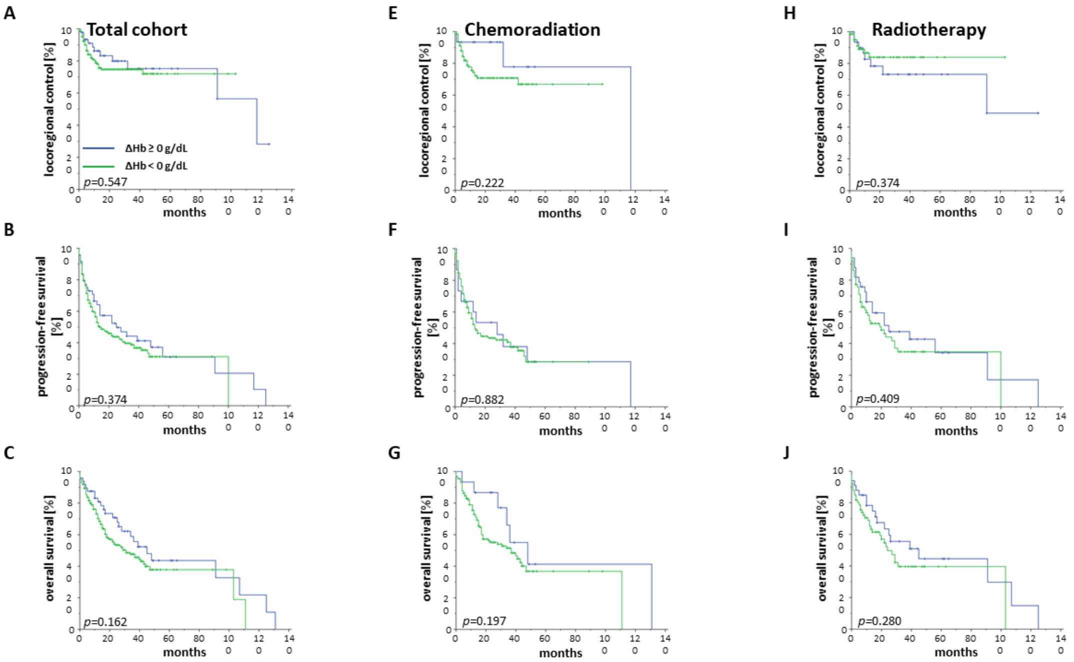

Supplementary Figure 3. Kaplan-Meier curves for LRC, PFS and OS in dependence of the peri-therapeutic hemoglobin change. (**A-C**) Kaplan-Meier curves for the entire elderly HNSCC cohort. (**D-F**) Kaplan-Meier curves for the chemoradiation group. (**G-I**) Kaplan-Meier curves for the radiotherapy group. *P*-values of log-rank tests are shown.

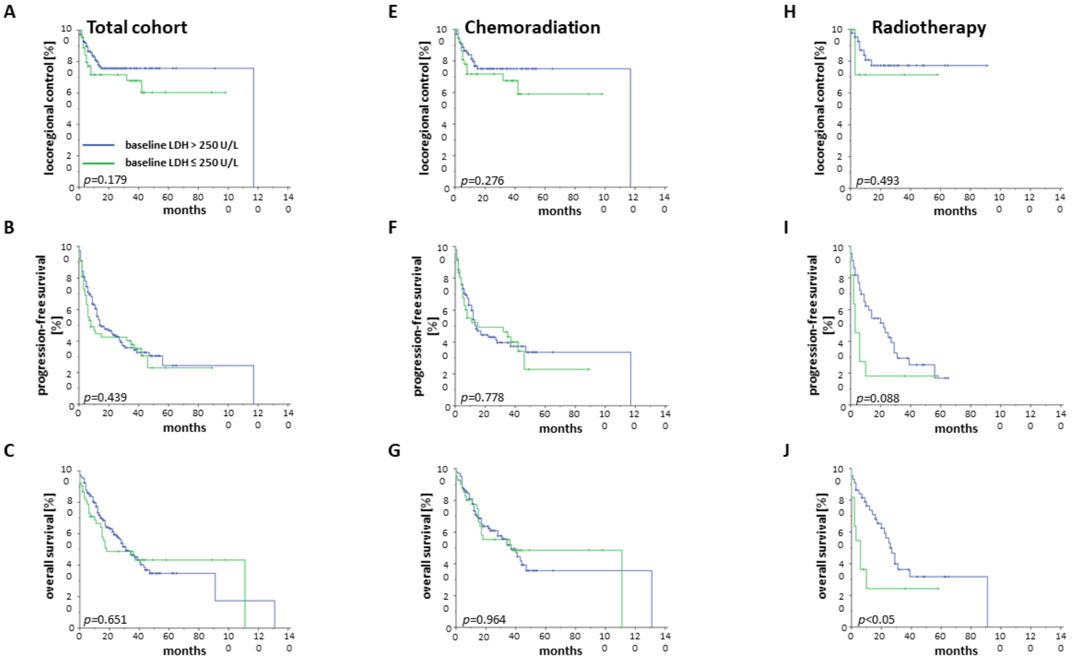

Supplementary Figure 4. LRC, PFS and OS stratified by the baseline LDH concentration. (**A-C**) Kaplan-Meier curves for the entire cohort with existing LDH values (n=199). (**D-F**) Kaplan-Meier curves of patients undergoing chemoradiation with accessible LDH values (n=144). (**G-I**) Kaplan-Meier curves of patients who were treated with radiotherapy and exhibited available LDH values (n=55). Log-rank-tests were performed for comparisons.

Table S1. Treatment details for (chemo)radiotherapy of elderly HNSCC patients (*n* = 246). Chemotherapy completion was assumed, if patients received at least 200 mg/m² cisplatin or 450 mg/m² carboplatin.

**Radiation therapy**	***n***	**%**
completed	213	86.6
discontinued	33	13.4
definitive	166	
completed	141	84.9
adjuvant	80	
completed	72	90.0
adjuvant		
mean radiation dose	60.2 Gy	
mean single dose	2.0 Gy	
definitive		
mean radiation dose	65.4 Gy	
mean single dose	2.0 Gy	
Chemotherapy	***n***	%
planned	147	
completed	109	74.1

Table S2. Toxicity results after (chemo)radiotherapy of elderly patients with HNSCC according to the Common Terminology Criteria for Adverse Events (CTCAE) v5.0.

**Acute**	***n***	%
CTCAE ≤ 2	108	43.9
CTCAE 3 – 4	138	56.1

CTCAE 5	0	0
**Chronic**		
CTCAE 0	31	13.7
CTCAE 1 – 2	150	66.4
CTCAE 3 – 4	45	19.9
CTCAE 5	0	0

Table S3: Toxicity results consisting various (chemo)radiotherapy-related adverse reactions according to the Common Terminology Criteria for Adverse Events (CTCAE) v5.0.

	**CTCAE grade**					
**Acute**	**0**	**1**	**2**	**3**	**4**	**5**
**dermatitis**	76	63	83	19	0	0
**dysphagia**	52	36	75	80	0	0
**nausea**	182	14	23	22	0	0
**mucositis**	66	32	98	46	0	0
**xerostomia**	127	42	69	4	0	0
**hoarseness**	202	19	18	3	0	0
**dyspnea**	217	12	9	4	0	0
**dysgeusia**	117	29	96	0	0	0
**pain**	108	35	71	28	0	0
**cytopenia**	44	51	82	67	2	0
**weight loss**	41	57	37	9	0	0
**acute kidney injury**	236	0	0	10	0	0
**Chronic**	**0**	**1**	**2**	**3**	**4**	**5**
**skin toxicity**	184	28	7	0	0	0
**dysphagia**	107	27	54	31	0	0
**nausea**	201	4	4	2	0	0
**mucositis**	186	17	12	3	0	0
**xerostomia**	72	62	81	3	0	0
**hoarseness**	193	19	6	0	0	0
**dyspnea**	205	7	4	2	0	0
**dysgeusia**	102	54	62	0	0	0
**pain**	170	18	26	4	0	0
**cytopenia**	205	5	5	1	0	0
**renal insufficiency**	204	5	5	4	0	0
**jaw and dental injuries**	181	6	20	10	0	0

© 2020 by the authors. Licensee MDPI, Basel, Switzerland. This article is an open access article distributed under the terms and conditions of the Creative Commons Attribution (CC BY) license (http://creativecommons.org/licenses/by/4.0/).
